# NGSmethDB: an updated genome resource for high quality, single-cytosine resolution methylomes

**DOI:** 10.1093/nar/gkt1202

**Published:** 2013-11-22

**Authors:** Stefanie Geisen, Guillermo Barturen, Ángel M. Alganza, Michael Hackenberg, José L. Oliver

**Affiliations:** ^1^Facultad de Ciencias, Departmento de Genética, Universidad de Granada, 18071-Granada, Spain and ^2^Laboratorio de Bioinformática, Instituto de Biotecnología, Centro de Investigación Biomédica, 18100-Granada, Spain

## Abstract

The updated release of ‘NGSmethDB’ (http://bioinfo2.ugr.es/NGSmethDB) is a repository for single-base whole-genome methylome maps for the best-assembled eukaryotic genomes. Short-read data sets from NGS bisulfite-sequencing projects of cell lines, fresh and pathological tissues are first pre-processed and aligned to the corresponding reference genome, and then the cytosine methylation levels are profiled. One major improvement is the application of a unique bioinformatics protocol to all data sets, thereby assuring the comparability of all values with each other. We implemented stringent quality controls to minimize important error sources, such as sequencing errors, bisulfite failures, clonal reads or single nucleotide variants (SNVs). This leads to reliable and high-quality methylomes, all obtained under uniform settings. Another significant improvement is the detection in parallel of SNVs, which might be crucial for many downstream analyses (e.g. SNVs and differential-methylation relationships). A next-generation methylation browser allows fast and smooth scrolling and zooming, thus speeding data download/upload, at the same time requiring fewer server resources. Several data mining tools allow the comparison/retrieval of methylation levels in different tissues or genome regions. NGSmethDB methylomes are also available as native tracks through a UCSC hub, which allows comparison with a wide range of third-party annotations, in particular phenotype or disease annotations.

## INTRODUCTION

DNA methylation is an epigenome mark involved in key biological processes (1–3), such as embryonic development, transcription, genomic imprinting, learning, memory or age-related cognitive decline (4–7). DNA methylation plays an important role in the origin and function of CpG islands (CGIs). Aberrant methylation (mostly hypermethylation) of CGIs has been implicated in the appearance of several disorders, such as cancer, immunodeficiency or centromere instability (8–14).

Many different techniques are available for DNA methylation profiling (15,16). Region-wide methods detect the methylation states of known CGIs or unmethylated fragments using either enzyme digestion or inmunoprecipitation, but frequently only ‘mean values’ of the corresponding regions can be derived from these methods. The advent of next-generation sequencing (NGS), together with bisulfite conversion of DNA, allows the generation of whole genome methylation maps at single-cytosine resolution (17–19). This provides an opportunity for studying important biological phenomena, such as the absence of methylation in a particular genome region over a range of tissues, the differential tissue methylation or the changes occurring along pathological conditions.

Several methylation databases centered in gene loci (20–23), tissues (24,25) or diseases (26–28) have been compiled. However, a wide variety of methodologies to pre-process the data, aligning the reads or inferring the methylation states has been used in compiling these databases, thus leading to methylomes obtained with very different methods or parameter sets to be included into the same database, which can bias downstream analyses. Additional problems are the regional resolution or the partial coverage of only some specific genome regions, which makes it difficult to use these data for comparative analyses. However, the single-base whole-genome methylomes stored in the new version of the ‘NGSmethDB’ database are all obtained using the same set of programs/scripts, and derived under the same settings and quality controls, thus allowing consistent comparative analyses of whole-genome methylomes.

## NGSmethDB CONTENT

Publicly available short-read data sets from NGS bisulfite-sequencing projects for different cell lines, fresh tissues and pathological tissues were downloaded mainly from NCBI GEO (29). An updated list of the data sets used for each genome, with detailed information on the source cell-line or tissue, is maintained online (http://bioinfo2.ugr.es/NGSmethDB/database.php).

To date, the database includes 87 methylome maps generated for CpG and CpHpG (H = A,C,T) sequence contexts in five different species for the most recent genome assembly: *Homo sapiens* (hg19), *Pan troglodytes* (panTro4), *Macaca mulatta* (rheMac3), *Mus musculus* (mm10) and *Arabidopsis thaliana* (tair10). The number of available methylomes by species was also increased: *Homo sapiens* (17), *Pan troglodytes* (5), *Macaca mulatta* (6), *Mus musculus* (30) and *Arabidopsis thaliana* (18). We restructured the database allowing the easy incorporation of novel species and/or methylomes, which ensures that the database will be always well-curated and maintained.

## EPIGENOME-WIDE METHYLOME MAPS

**Figure 1.**
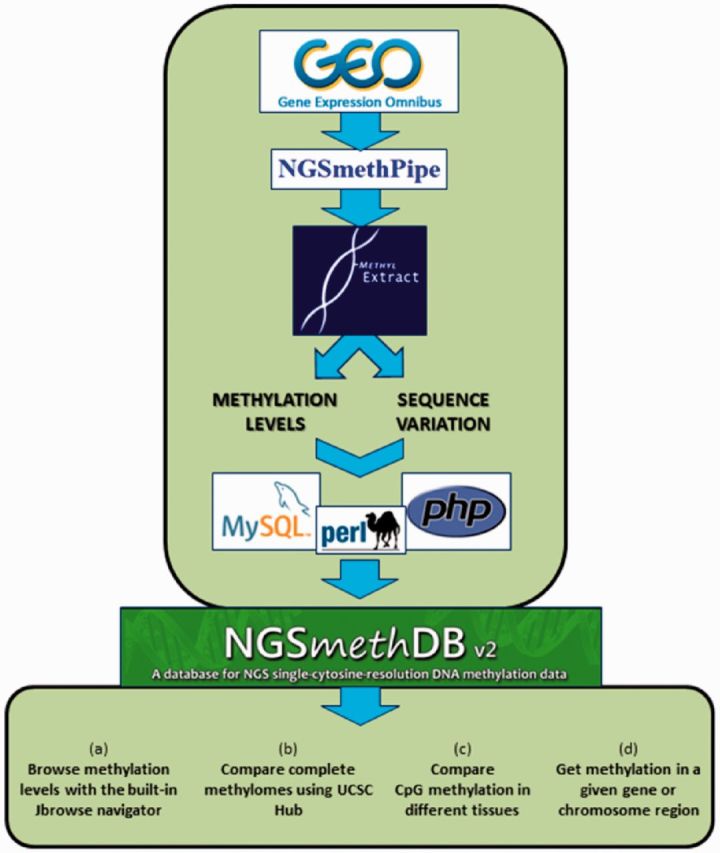
Flow diagram showing the implemented steps and main features of NGSmethDB.

A flow diagram delineating the implementation and main features of NGSmethDB is shown in Figure 1. Short-read data sets were pre-processed and aligned to the corresponding reference genome using ‘NGSmethPipe’ (31), and then profiling the methylation levels by means of ‘MethylExtract’ (32).

### Alignment of short-reads

NGSmethPipe (http://bioinfo2.ugr.es/NGSmethPipe/) implements several pre-processing steps to improve the alignment quality, like the trimming prior to the adapter detection. It uses ‘Bowtie’ (33) as an external aligner applied on a three-letter alphabet. To map a higher number of reads without compromising the mapping quality, NGSmethPipe uses a ‘seed extension’ method applied to the Bowtie alignments, similar to that used in ‘miRanalyzer’ (34,35). Short-read alignment per se is a highly parameterized process. Adding the NGSmethPipe-specific parameters results in obtaining a notable parameter space. Relaxed parameters will lead to a higher coverage (i.e. many cytosines can be profiled), but a higher number of incorrect alignments can also be expected. On the contrary, strict parameters might lead to a lower coverage, thereby discarding a considerable amount of valuable information. For the presented database, we carried out a careful study to measure alignment accuracy as a function of the seed length and number of mismatches to obtain the best parameter set. NGSmethPipe now uses these settings as default options (see the ‘Quick start’ section in http://bioinfo2.ugr.es/NGSmethPipe/Manual.html for a complete list of defaults).

### Methylation profiling

For the methylation profiling carried out by MethylExtract (http://bioinfo2.ugr.es/MethylExtract/), we implemented a number of stringent quality controls, carefully chosen to minimize important error sources [see (32) for a complete description]:

1. A first potential error source in methylation profiling is the bisulfite conversion failure. In modern protocols, usually <1% of all unmethylated cytosines fail to be converted by bisulfite treatment. Thus, some positions are incorrectly profiled, i.e. some inferred methylcytosines are actually unmethylated. To cope with this error, we first implemented (as an option) a method proposed by Lister (17) to detect reads with a high number of unconverted cytosines: if this option is activated, the reads with at least 90% of unconverted cytosines in non-CpG contexts were eliminated. Second, when a non-methylated genome is available (e.g. the chloroplast genome for *Arabidopsis* data sets), MethylExtract can associate a *P*-value, based on binomial statistics, and a false discovery rate to the extracted methylation levels [see (32) for details]. For the sake of uniformity, and given the lack of non-methylated genomes for all the included species, we do not use this feature in populating NGSmethDB. However, when using the data mining tools, the user can choose the minimum coverage required for a cytosine methylation context. In addition, the methylation browser shows all the individual methylation values.
2. Other potential sources for incorrect methylation profiling are sequencing errors. We used the assigned Phred score (36) to limit the contribution of incorrectly sequenced bases. By setting Q ≥ 20, we are only accepting bases with a *P* < 0.01 to be incorrectly called.
3. In methylation profiling, SNVs are probably the most disregarded error source. Over two-thirds of all SNPs occur in a CpG context, having two alleles: C/T or G/A (37). Most other tools would interpret a C > T substitution as an unmethylated cytosine, although a certain number of them are actually SNVs, and therefore the inference would be wrong. A C/T SNV manifests on the complementary DNA strand as an adenine, while bisulfite deamination does not affect the guanine on the complementary strand (38). We take advantage of this observation to detect putative SNVs by means of a threshold method based on VarScan, thus avoiding subsequent erroneous methylation profiling.
4. Duplicated (clonal) reads provoked by the polymerase chain reaction step adds another layer of potential errors in methylation profiling. MethylExtract implements an option to delete duplicated reads without eliminating meaningful biological information. In populating NGSmethDB, we used this option of MethylExtract.
5. Lastly, when needed, we carried out 5′ end trimming of reads. As implemented in ‘Bismark’ (39), the first N nucleotides are removed from the 5′ end of the read (3 nt in case of the MspI restriction sites of the reduced representation bisulfite sequencing protocol).

### Methylome maps

The resulting high-quality methylomes, obtained under uniform settings as indicated earlier in the text, were stored in a ‘MySQL’ database back-end, which is used to serve visualization, data mining and database dumps. Methylation maps for minimum coverages of 1, 3, 5 or 10 reads (http://bioinfo2.ugr.es/NGSmethDB/database.php) were generated. We used ‘Perl’ scripts to automate data parsing and database management.

An outstanding feature of MethylExtract is the calling of SNVs from the same sequence library of bisulfite-treated DNA used to infer methylation states. Therefore, besides methylation tracks, SNV tracks were also generated for each sample and made available for download or visualization through the methylation browser.

## THE METHYLATION BROWSER

**Figure 2.**
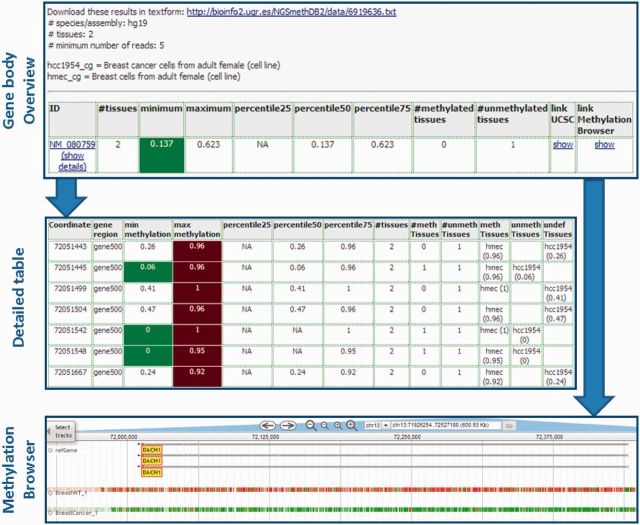
Gene hypomethylation in the *DACH1* tumor suppressor gene. The figure shows the average CpG methylation in the gene body (Gene body Overview), the methylation levels at single cytosines (detailed table) and its visualization in the methylation browser for normal (*hmec*) and cancer (*hcc1954*) breast cell lines. Average and single-base CpG methylation levels can be downloaded for further analysis. Short-read samples GSM721195 HMEC-methylC-Seq and GSM721194 HCC1954-methylC-Seq (42), downloaded from GEO (29), were used to generate the corresponding methylome maps.

The user interface was improved by replacing ‘Gbrowse’ with ‘Jbrowse’ (40,41), resulting in a methylation browser with a fast and smooth scrolling and zooming mechanism (Figure 2). This speeds data download and upload, and requires light server resources.

Users can include their own data in ‘bigWig’, ‘VCF’, ‘gff’ or ‘bed’ formats (https://genome.ucsc.edu/FAQ/FAQformat.html), thus comparing their data directly with the NGSmethDB methylomes. User data sets are not uploaded to the server, but instead opened directly via the Java interface. This ensures a quick and stable data integration without compromising the server stability and response time.

RefSeq (30) gene names were indexed, thus making them searchable via the browser interface. In addition, NGSmethDB includes many other annotation tracks (CpGislands, promoters, SNPs, repeats, isochores, phastCons) that can be viewed and compared with the methylation maps.

A detailed manual (http://bioinfo2.ugr.es/NGSmethDB/manual.php) guides the user through the different steps to quickly browse the Web site and download NGSmethDB methylation maps. Furthermore, a general and context-dependent help about searching, moving, zooming and showing/hiding tracks with JBrowse has been interactively integrated in the proper methylation browser window.

## A UCSC TRACK HUB FOR NGSmethDB METHYLOMES

We also made NGSmethDB methylation maps directly available through a UCSC track hub, a web-accessible directory of genomic data that can be viewed on the UCSC genome browser (http://genome.ucsc.edu/goldenPath/help/hgTrackHubHelp.html). Therefore, high-quality NGSmethDB methylomes can be visualized and tuned on the UCSC genome browser as native tracks. This allows the comparison with a wide range of third-part annotations, in particular phenotype and disease associations, or the ENCODE annotation tracks.

## DATA MINING TOOLS

Similar to the first version of NGSmethDB, the user interface was based on the practical appeals of epigenome-wide analysis: namely, the possibility to (i) obtain methylation values for particular chromosomal regions or tissues, (ii) analyze promoter methylation for a set of tissues and (iii) compare methylation patterns across a set of different tissues. To this end, three different database mining tools were developed to allow the user to filter, compare, analyze and download the methylation data in different species, tissues, developmental stages or diseases:

1. Comparison of cytosine methylation levels in different tissues. The user can select the sequence context (CG or CHG) and the methylation states for comparison: methylated versus unmethylated, methylated versus intermediate, unmethylated versus intermediate or all of them.
2. The methylation states of different gene regions, including gene body, promoters, 3′ ends, exons and introns, can be retrieved/downloaded.
3. Methylation data for single cytosines within a given chromosome region can be retrieved/downloaded; a detailed table is provided with direct links to our methylation browser and the UCSC genome browser.

New features in this version of the database are the possibility to supply a customized set of regions in bed format (https://genome.ucsc.edu/FAQ/FAQformat.html) to obtain the methylation levels or a gene list to retrieve the data in a given gene region. Depending on the amount of requested data (mainly, the number of tissues), some of these tools might take several hours to process the requested data. To overcome this limitation, we implemented PHP sessions (http://php.net/manual/en/ref.session.php), thus offering the user the possibility to submit >1 job at a time. An ID is assigned to each submitted job. Running jobs are shown under the header ‘running’, providing the possibility to also cancel the jobs. Once finished, a long life link becomes available, allowing the user to retrieve the results within 30 days. If there are >5 jobs running from the same user, the next job gets queued and will be executed automatically as soon as the previous job has finished.

## WORKING EXAMPLES

As a first example, the hypomethylation of the *DACH1* tumor suppressor gene (42) was analyzed by means of NGSmethDB. Human *DACH1* on chromosome 13 encodes a chromatin-associated protein that associates with other DNA-binding transcription factors to regulate gene expression and cell fate determination during development. Figure 2 shows the results when analyzing the gene body methylation of this gene for normal (*hmec*) and cancer (*hcc1954*) breast cell lines. NGSmethDB first shows a summary statistics of the methylation levels across the used set of tissues (Figure 2, top), also providing links to a table with detailed methylation levels at single cytosine resolution (Figure 2, middle) and its visualization in the methylation browser (Figure 2, bottom). A global gene hypomethylation in breast cancer, as compared with healthy tissue, can be clearly appreciated.

**Figure 3.**
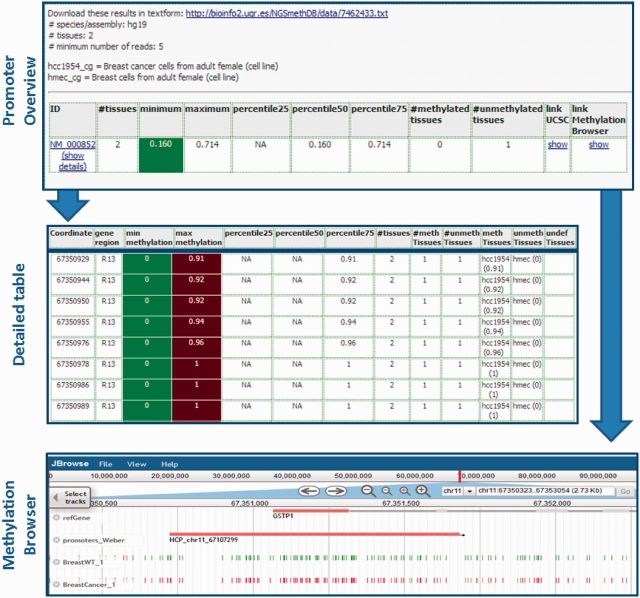
*GSTP1* hypermethylation in breast cancer. *GSTP1* codes for the glutathione S-transferase Pi-1. The screenshot of the NGSmethDB methylation browser (bottom) corresponds to positions 67349906–67356735 of the human chromosome 11. The promoter region, as defined in ref. (44), and the NGSmethDB methylation maps for normal (*hmec*) and cancer (*hcc1954*) breast cell lines are shown. The healthy breast promoter appears as unmethylated (green vertical bars), whereas the breast cancer tissue is heavily methylated (red vertical bars). Some rows of the detailed methylation table at single cytosines with coverage of at least five reads are shown (middle).

A second example shows the analysis of the hypermethylation of the *GSTP1* promoter in cancer. This gene codes for the glutathione S-transferase Pi-1, an enzyme involved in cellular detoxification of xenobiotics and carcinogens, being a promising biomarker for cancer diagnosis and prognosis (43). The methylation map of the promoter region in normal and cancer breast tissue provided by NGSmethDB is shown in Figure 3 (bottom). A detailed table with methylation values at individual CpGs is shown in Figure 3 (middle). NGSmethDB analysis clearly shows the hypermethylation of this promoter region in breast cancer.

Lastly, NGSmethDB methylomes have been used to compile ‘CpGislandEVO’ (45), a specialized genome platform for the comparative evolutionary genomics of CGIs. Both databases may be useful for studies relating DNA methylation and the evolutionary rates of different genome elements (46).

## CONCLUSIONS

NGSmethDB provides high-resolution epigenome-wide methylome maps for a collection of the best-assembled eukaryotic genomes. All methylome maps stored in the database were obtained under uniform conditions, i.e. using strictly the same bioinformatics protocol for all raw data sets including the same parameter settings and the same stringent quality controls. SNV variants, obtained jointly with methylation values, have also been provided as accompanying tracks, which may facilitate to analyze the relation between DNA methylation and sequence variation. To widen comparative studies, the NGSmethDB methylome maps are connected to a UCSC track hub, thus allowing the comparison to third-part phenotype or disease annotation tracks.

## FUNDING

Spanish Government [BIO2008-01353 to J.L.O. and BIO2010-20219 to M.H.], and Basque country ‘AE’ grant (to G.B.). Funding for open access charge: Department of Genetics, University of Granada, Spain.

*Conflict of interest statement*. None declared.
